# Supplementary material for: The causal relationship between the human gut microbiota and pyogenic arthritis: a Mendelian randomization study
Source: Front Cell Infect Microbiol. 2024 Nov 26;14:1452480. doi: 10.3389/fcimb.2024.1452480 (PMC11629706; doi:10.3389/fcimb.2024.1452480)
Supplement: Supplementary file 1 [file DataSheet1.zip › mendelian test/ebi-a-GCST90027840.csv_forest.pdf]

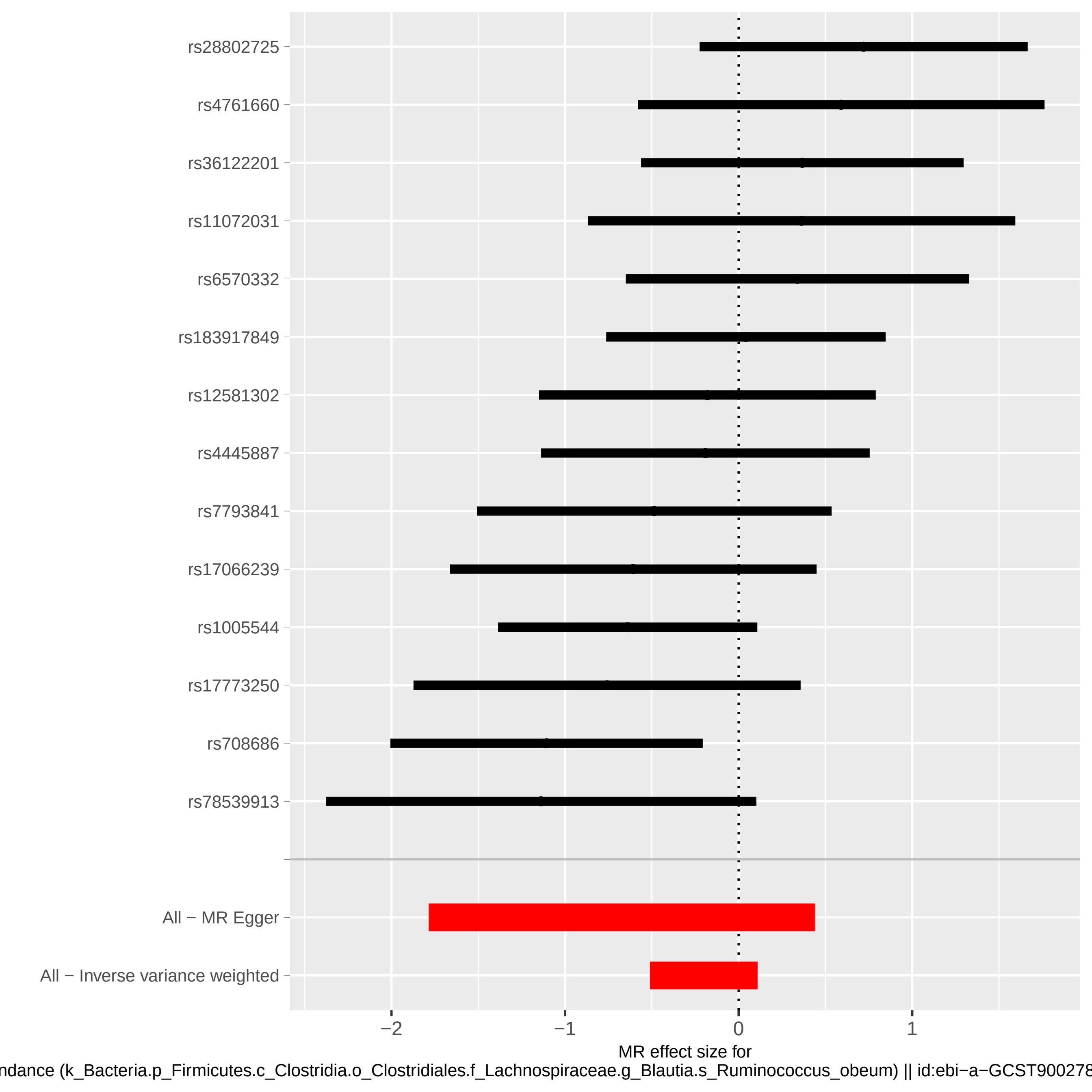
rs28802725
rs4761660
rs36122201
rs11072031
rs6570332
rs183917849
rs12581302
rs4445887
rs7793841
rs17066239
rs1005544
rs17773250
rs708686
rs78539913
All − MR Egger
All − Inverse variance weighted
−2
−1
0
1
MR effect size for
ndance (k_Bacteria.p_Firmicutes.c_Clostridia.o_Clostridiales.f_Lachnospiraceae.g_Blautia.s_Ruminococcus_obeum) || id:ebi−a−GCST900278
